# Predictive value of placental real-time shear wave elastography combined with 3-dimensional power Doppler index for preeclampsia

**DOI:** 10.1097/MD.0000000000037372

**Published:** 2024-03-08

**Authors:** Fei Tian, Lian-feng Dou, Li-wei Tang, Qi-min Gao, Bao-wei Li

**Affiliations:** a Ultrasound Department, Binzhou Medical University Hospital, Binzhou, Shandong Province, China; b Emergency Department, Binzhou Medical University Hospital, Binzhou, Shandong Province, China.

**Keywords:** preeclampsia, real-time shear wave elastography, three-dimensional power Doppler index, ultrasonography

## Abstract

This study aimed to investigate the value of placental real-time shear wave elastography combined with three-dimensional power Doppler index (3D-PDI) in the prediction of preeclampsia. We conducted a retrospective study selecting 60 pregnant women diagnosed with preeclampsia as the experimental group and 60 normal pregnant women as the control group from January 2021 to December 2022. The elastic modulus values of different regions of the placenta and placental 3D-PDI were detected and compared between the two groups. The ROC curve was used to evaluate the diagnostic value of each parameter, alone or in combination, for preeclampsia. The study findings demonstrated that the elastic modulus values of different regions of the placenta and 3D-PDI of the two groups have statistical significance. The values of SWE, VI, FI, and VFI are different in prediction of preeclampsia, and the combination of various parameters can improve the prediction value. Overall, our study provides a valuable method for the prediction of preeclampsia with the advantages of non-invasiveness, efficiency, and simplicity.

## 1. Introduction

Preeclampsia (PE) is a unique complication of women during pregnancy, which refers to high blood pressure that develops after 20 weeks of pregnancy with proteinuria or without proteinuria but with new onset of any of the following: thrombocytopenia, renal insufficiency, impaired liver function, pulmonary edema, or headache unresponsive to medication.^[1,2]^ Besides, PE can cause headache, dizziness, nausea, vomiting, epigastric discomfort, and other symptoms. PE is the leading cause of maternal morbidity and mortality worldwide with the incidence of 3% to 8%.^[3]^ Until now, the pathogenesis of PE is not clear. It is believed that the failure of uterine spiral artery recasting causes placental ischemia and hypoxia dysplasia, a variety of placental factors entering the maternal blood leads to systemic inflammatory response and vascular endothelial damage, and finally, PE is developed.^[4]^ It has also been suggested that placental stiffness increases in patients with PE for the following reasons: The mechanisms for increased placental stiffness in PE may be that trophoblast invasion of the maternal spiral arteries is impaired, resulting in reduced placental perfusion and in a hypoxic placental milieu. Hypoxia arouses collagen and fibrin deposition and fibrosis, which together leads to increased placental stiffness.^[2,5,6]^ Postpartum PE patients can return to normal, so the abnormality of the placenta plays a very important role in the occurrence of PE. Therefore, placental monitoring is of great significance to understand fetal growth and development and take effective clinical treatment in a timely manner.^[7]^ The diagnosis of PE in the past requires the detection of blood pressure, urine protein, and clinical symptoms of the patient, but not all pregnant women can be tested for the above relevant indicators in time, so that they are only found when they have serious complications, which seriously affects the health of the mother and fetus. At present, there are few indicators that can predict PE, and there is a lack of effective predictors in the first trimester. In particular, there is a lack of effective joint predictors. However, noninvasive and inexpensive character of ultrasound examination has enabled the use of ultrasound for very early detection (in the first and the second trimester) of pregnancy-specific diseases, such as gestational diabetes and PE.^[8–10]^ Women with hypertensive disorders of pregnancy have increased shear wave elastography (SWE) of the placenta compared with the placentas of women with uneventful pregnancies.^[11]^ Therefore, we aimed to evaluate the tissue stiffness of the placenta and the placental blood perfusion, respectively, to evaluate the predictive value of these 2 parameters for PE, alone or in combination.

## 2. Materials and methods

### 2.1. Research subjects

From January 2021 to December 2022, 60 pregnant women who were treated in our hospital and diagnosed with PE were selected as the experimental group, and 60 normal pregnant women in the same period were selected as the control group. All participants were singletons, without fetal malformations, uterine malformations, underlying medical conditions, and other pregnancy comorbidities. All maternal placentas were located on the anterior wall of the uterus and can be fully and clearly monitored, which is helpful to ensure the accuracy of the measurement. PE diagnostic criteria are as follows: systolic blood pressure of 140 mm Hg or more or diastolic blood pressure of 90 mm Hg or more on 2 occasions at least 4 hours apart after 20 weeks of gestation in a woman with a previously normal blood pressure; systolic blood pressure of 160 mm Hg or more or diastolic blood pressure of 110 mm Hg or more. (Severe hypertension can be confirmed within a short interval [minutes] to facilitate timely antihypertensive therapy.) Proteinuria 300 mg or more per 24 hours of urine collection (or this amount extrapolated from a timed collection) or protein/creatinine ratio of 0.3 mg/dL or more or Dipstick reading of 2+ (used only if other quantitative methods not available). Or in the absence of proteinuria, new-onset hypertension with the new onset of any of the following: thrombocytopenia: platelet count less than 100,000 × 10^9^/L; renal insufficiency: serum creatinine concentrations greater than 1.1 mg/dL or a doubling of the serum creatinine concentration in the absence of other renal disease; impaired liver function: elevated blood concentrations of liver transaminases to twice normal concentration; pulmonary edema; new-onset headache unresponsive to medication and not accounted for by alternative diagnoses or visual symptoms.^[1]^ The age, height, weight, gestational age, reproductive history, and history of PE of all study participants were recorded to calculate the body mass index (BMI). This experiment has been approved by the Research Ethics Committee of Binzhou Medical University Hospital, and all study subjects received informed consent (Ethics approval number: 2023-LW-69).

### 2.2. Research methods

#### 2.2.1. Detection of placental elasticity.

**Figure 1.**
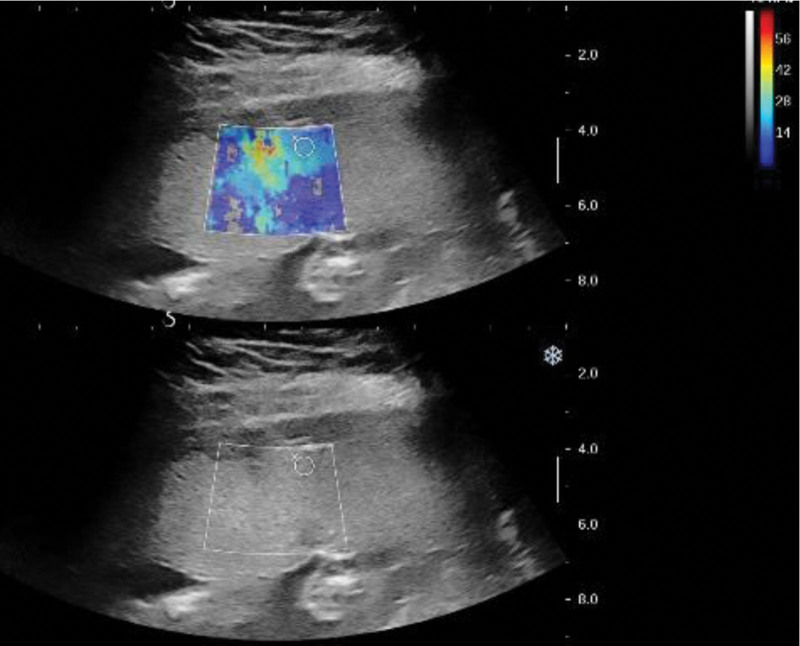
Detection of placental elasticity.

Sonologist Aixplorer V color Doppler ultrasound diagnostic instrument was equipped with convex array probe (frequency 3.5–5.0 MHz) and SWE quantitative analysis system. The pregnant woman lay on her back. Routine ultrasound examination determines the center and edge of the placenta. The probe was gently placed vertically and fixed at the scanning site. The pregnant woman was instructed to breathe calmly. Fetal movements and uterine contractions were avoided. Placental umbilical cord insertion point and obvious calcification and blood sinus were avoided. In elastography mode, when the image is stable, the quantitative analysis system was performed. The SWE value was obtained with the diameter of the sampling frame at 5 mm and the sampling depth was controlled within 8 cm. The average value of 3 measurements with 3- to 5-second intervals was calculated for each region of interest (Fig. 1).

#### 2.2.2. Detection of placental 3-dimensional power Doppler index.

**Figure 2.**
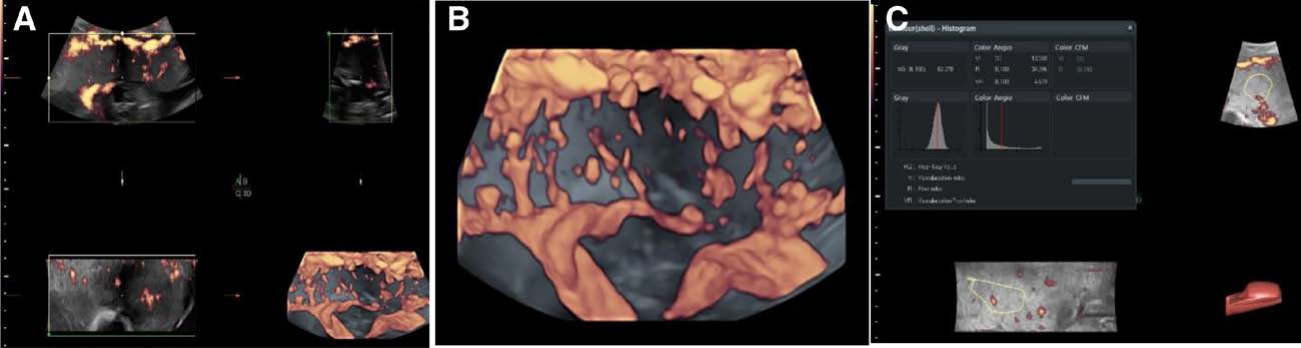
Detection of placental 3-dimensional power Doppler index. (A) Volumetric data acquisition; (B) Three-dimensional image of placental blood vessels; (C) VOCAL software quantitative analysis. VOCAL = virtual organ computer-aided analysis.

GE Voluson E8 color Doppler ultrasound diagnostic instrument was equipped with abdominal 3-dimensional volumetric probe (frequency 4.0–8.0 MHz) capable of 2-dimensional, 3-dimensional, and 3-dimensional energy Doppler blood flow imaging, and virtual organ computer-aided analysis (VOCAL) software. During the examination, the pregnant woman was instructed to breathe calmly. Fetal movements were avoided. The entrance of the umbilical cord of the placenta was selected for measurement in the 3-dimensional energy Doppler vascular mode. All placental thickness tissue were enveloped, and the volume data were collected quickly. VOCAL software was used to obtain the placenta three-dimensional power Doppler index (3D-PDI): VI, FI, VFI. All data collected in this study were performed by an experienced prenatal diagnostic sonographer (Fig. 2).

### 2.3. Statistical analysis

Using SPSS 26.0 statistical software, the measurement data are expressed as mean ± standard deviation (*x̄* ± *s*), and the mean comparison of the 2 samples is measured by the *t*-test of 2 independent samples, and the counting data are expressed in n (%), using the χ^2^ test. The ROC was used to evaluate the predictive value of each parameter, alone or in combination, for PE, and to calculate the incidence of PE at a false-positive rate of 5%. *P* < .05 is statistically significant.

## 3. Results

### 3.1. Comparison of general data of pregnant women in the experimental and control group

There was no significant difference in age, gestational age, BMI, history of PE, and history of menstrual labor between the 2 groups (all *P *> .05; Table 1).

**Table 1:** Comparison of general information on pregnant women in the 2 groups.

Group	Case number	Age (year *x̄* ± *s*)	Gestational age (week *x̄* ± *s*)	BMI (kg/m^2^ *x̄* ± *s*)	Preeclampsia history (n [%])	Birth history (n [%])
Experimental group	60	33.08 ± 5.76	32.08 ± 2.92	22.15 ± 1.78	20 (33.33)	26 (43.33)
Control group	60	32.90 ± 5.90	31.97 ± 2.89	22.05 ± 1.78	12 (20.00)	18 (30.00)
*t*/*X*^2^ value *P* value		0.172 .864	0.220 .826	0.293 .770	2.727* .099	2.297* .130

BMI = body mass index.

*represents the values obtained by χ^2^ test.

### 3.2. Comparison of placenta SWE values of pregnant women in experimental and control group

The SWE values of central fetal face, central maternal surface, marginal fetal surface, and marginal maternal surface of placenta in the experimental group were higher than those in the control group, and the differences were statistically significant (all *P* < .05). However, there was no difference in SWE values in different regions of the placenta in each group (all *P *> .05; Table 2).

**Table 2:** Comparison of placental elastic modulus values in different regions of the 2 groups (***x̄*** ± *s*).

Group	Case number	Central fetal surface	Central maternal surface	Marginal fetal surface	Marginal maternal surface	Average value
Experimental group	60	8.24 ± 1.45	8.42 ± 1.63	8.09 ± 1.32	8.01 ± 1.35	8.19 ± 1.41
Control group	60	6.79 ± 0.92	6.74 ± 0.91	6.80 ± 0.89	6.72 ± 0.95	6.76 ± 0.81
*t* value *P* value		6.547 .000	6.947 .000	6.273 .000	6.009 .000	6.797 .000

### 3.3. Comparison of placenta 3D-PDI of pregnant women in experimental and control group

The placenta VI, FI, and VFI of the experimental group were lower than those in the control group, and the differences were statistically significant (all *P *< .05; Table 3).

**Table 3:** Comparison of three-dimensional power Doppler indices of 2 groups of placenta (***x̄*** ± *s*).

Group	Case number	VI	FI	VFI
Experimental group	60	17.14 ± 2.12	40.56 ± 2.94	6.88 ± 1.34
Control group	60	19.77 ± 2.47	43.94 ± 3.98	9.08 ± 2.18
*t* value *P* value		−6.250 .000	−5.300 .000	−6.669 .000

FI = blood flow index, VFI = vascularization-blood flow index, VI = vascularization index.

### 3.4. Relationship between PE and maternal placental SWE value and 3D-PDI alone or in combination

**Figure 3.**
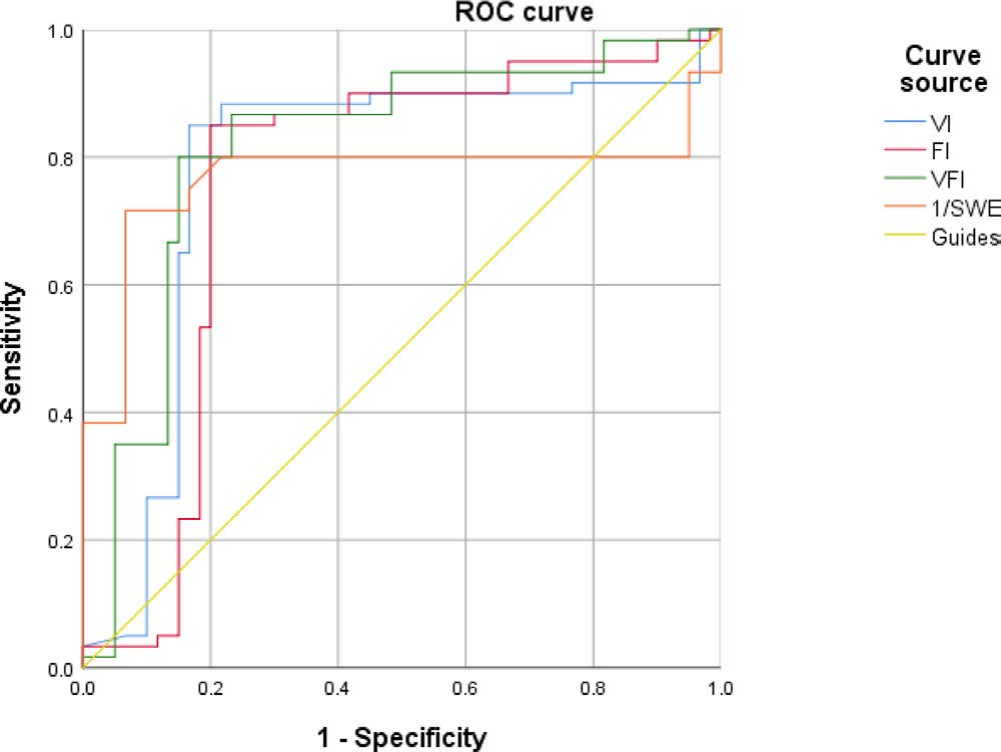
ROC curves for predicting preeclampsia, respectively. FI = blood flow index, ROC = receiver operating characteristic curve, SWE = modulus of elasticity value, VFI = vascularization-blood flow index, VI = vascularization index.

The ROC was plotted, and the area under the ROC was calculated to compare the sensitivity and specificity of SWE, VI, FI, and VFI in predicting PE. The ROCs show that all of SWE, VI, FI, and VFI have predictive value for PE (all *P* < .05). When the false-positive rate is 5%, the sensitivity of SWE, VI, FI, and VFI is 0.800, 0.850, 0.850, and 0.800, respectively (Fig. 3).

**Figure 4.**
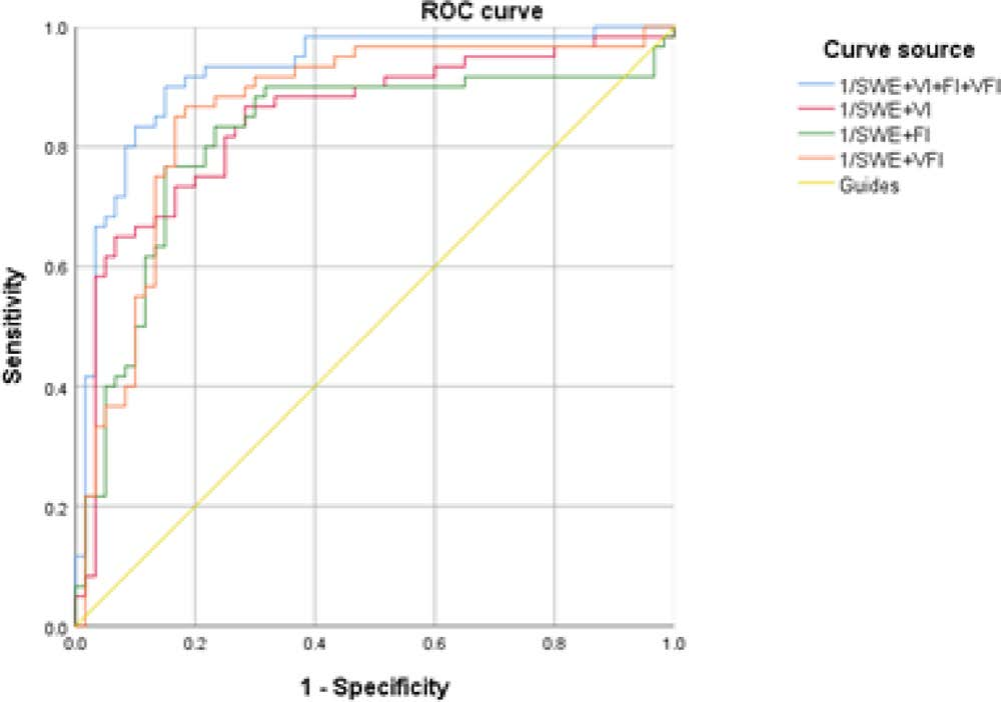
ROC curves for joint prediction of preeclampsia. FI = blood flow index, ROC = receiver operating characteristic curve, SWE = modulus of elasticity value, VFI = vascularization-blood flow index, VI = vascularization index.

The parameters were jointly combined to plot ROCs, and the SWE combined VI, FI, VFI and the combination of the 4 had high predictive value (all *P* < .05), and when the false-positive rate was 5%, the combined diagnostic sensitivity was 0.750, 0.800, 0.850, and 0.833, respectively (Table 4 and Fig. 4).

**Table 4:** Combined detection of area under the ROC of preeclampsia.

Variable	Area	Standard error	95% confidence interval	
			Lower limit	Upper limit
1/SWE + VI	0.846	0.037	0.772	0.919
1/SWE + FI	0.816	0.042	0.733	0.899
1/SWE + VFI	0.861	0.036	0.790	0.932
1/SWE + VI + FI + VFI	0.916	0.027	0.863	0.968

FI = blood flow index, ROC = receiver operating characteristic curve, SWE = modulus of elasticity value, VFI = vascularization-blood flow index, VI = vascularization index.

## 4. Discussion

SWE is mainly used to evaluate the hardness of lesions. SWE has been widely used in the diagnosis of breast, thyroid, liver, and other organs, as it not only avoids the subjective influence of the operator and comparison with surrounding tissues but also has the advantages of noninvasion and good repeatability.^[1]^ The use of SWE technology in obstetrics is still relatively rare, but it has been reported that elastography is safe for pregnant women.^[12]^ Three-dimensional power color Doppler ultrasound is a noninvasive ultrasound technique developed from conventional ultrasound to clearly show blood perfusion.^[13]^ It is not affected by the detection angle and is sensitive to low-speed blood flow and tiny blood vessels in the placenta.^[14]^ The reconstruction image is more intuitive and can be analyzed offline by VOCAL software to obtain VI, FI, VFI (VI represents the number of blood vessels in the area of interest; FI stands for area of interest blood flow strength; VFI is a comprehensive evaluation index representing the sum of blood vessels and blood flow), thereby quantitatively evaluating the number of vessels and blood flow rate of placental tissue.^[15]^ In this study, the combination of these 2 techniques was applied to evaluate placental function more comprehensively, meanwhile, the limitations and drawbacks of previous evaluation methods such as placental grading and color Doppler can be avoided.

There was no difference in the general information of pregnant women in experimental and control group, such as age, gestational age, BMI, etc, which avoids the interference of other factors other than PE on the study results. The elastic modulus values of different parts of the placenta in the experimental group were higher than those in the control group, and there was no difference in the elastic modulus values of different parts of the placenta in each group, which was consistent with the previous results.^[2,16–18]^ That is to say the selection of the region of interest for placental elasticity measurement does not affect the elastic value. The basic pathophysiological changes of PE are spasm of small blood vessels throughout the body, increased peripheral vascular resistance, decreased blood supply to the uterus placenta, resulting in placental vascular dysfunction, abnormal trophoblast structure, increased inflammation level, increased fibrin deposition, etc, and further resulting in changes in placental function and hardness.^[19–22]^ Traditional 2-dimensional ultrasound can only grade the placenta, provide placental morphological characteristics. It is interfered by many subjective factors, while SWE can objectively and quantitatively reflect the change of placental tissue hardness, and indirectly reflect the functional state of the placenta in patients with PE. The results of this study also suggest that the placental 3D-PDI VI, FI, and VFI of PE patients are lower than that of normal pregnant women in the control group. It is because that the blood pressure of pregnant women with PE is increased, and the placental circulatory pressure increases, resulting in irreversible embolism, stenosis, degeneration, necrosis, and other conditions in placental blood vessels, which usually worsen with the exacerbation of the disease.^[23,24]^ Compared with color Doppler ultrasound, 3-dimensional power Doppler can more clearly demonstrate the microvascular and low-speed blood flow in the placenta, quantitatively evaluate the thinning and quantity of blood vessels in the placenta, and is not affected by scanning angle, vascular aliasing, blood flow velocity, etc. It can more clearly demonstrate the placental blood perfusion status to determine whether there is ischemia and the degree of ischemia in the placenta in patients with PE.

By ROC analysis, we found that placental SWE, 3D-PDI VI, FI, and VFI all have the value in prediction of PE, and the area under the ROC was 0.769, 0.779, 0.754, and 0.817, respectively. In this study, we further evaluated whether the combination of multiple parameters could improve the predictive value for PE. The results showed that the area under the ROC of SWE values combined with VI, FI, or VFI, or the combination of the 4 were 0.846, 0.816, 0.861, and 0.916, respectively, which were higher than the predicted values of each parameter alone. This indicates that placental SWE, VI, FI, and VFI are helpful in predicting PE, and that the combination of multiple parameters can improve the prediction efficiency.

## 5. Conclusion

In conclusion, the results show that the placental elastic modulus value, 3D-PDI VI, FI, and VFI have different levels of predictive value for PE, and the combined detection of various parameters can improve the prediction value, and the combined prediction value of 4 parameters is the highest. SWE and 3D-PDI, as a noninvasive diagnostic method, can be used as an important means to detect placental function in patients with PE by detecting placental hardness and blood perfusion, and provide an objective basis for clinical diagnosis.

The shortcomings of this study are the lack of placental pathology examination information as a control, and there is no group discussion of the severity of PE. The elasticity measurement is limited by depth, and it is impossible to detect the elasticity of posterior wall and lateral wall placenta, which will also be the focus of our future research. We will also conduct a multi-center study, collect data from different research centers, and further explore the predictive value of placental elasticity and 3D-PDI alone or in combination for PE.

## Author contributions

**Conceptualization:** Fei Tian.

**Data curation:** Lian-feng Dou.

**Formal analysis:** Li-wei Tang.

**Investigation:** Bao-wei Li.

**Methodology:** Qi-min Gao.

## References

[1] Gestational hypertension and preeclampsia: ACOG practice bulletin, number 222. Obstet Gynecol. 2020;135:e237–60. 32443079 10.1097/AOG.0000000000003891

[2] Spiliopoulos M Kuo CY Eranki A. Characterizing placental stiffness using ultrasound shear-wave elastography in healthy and preeclamptic pregnancies. Arch Gynecol Obstet. 2020;302:1103–12. 32676857 10.1007/s00404-020-05697-x PMC7646518

[3] Ali Z Khaliq S Zaki S. Differential expression of placental growth factor, transforming growth factor-β and soluble endoglin in peripheral mononuclear cells in preeclampsia. J Coll Physicians Surg Pak. 2019;29:235–9. 30823949 10.29271/jcpsp.2019.03.235

[4] Bosco C González J Gutiérrez R. Oxidative damage to pre-eclamptic placenta: immunohistochemical expression of VEGF, nitrotyrosine residues and von Willebrand factor. J Matern Fetal Neonatal Med. 2012;25:2339–45. 22612323 10.3109/14767058.2012.695823

[5] Gilkes DM Bajpai S Chaturvedi P. Hypoxia-inducible factor 1 (HIF-1) promotes extracellular matrix remodeling under hypoxic conditions by inducing P4HA1, P4HA2, and PLOD2 expression in fibroblasts. J Biol Chem. 2013;288:10819–29. 23423382 10.1074/jbc.M112.442939 PMC3624462

[6] Nikitina ER Mikhailov AV Nikandrova ES. In preeclampsia endogenous cardiotonic steroids induce vascular fibrosis and impair relaxation of umbilical arteries. J Hypertens. 2011;29:769–76. 21330936 10.1097/HJH.0b013e32834436a7 PMC3053428

[7] Zhao YY Wang MY Dong YW. Correlation between uterine artery hemodynamics and placental perfusion parameters and preeclampsia in patients with hypertension during pregnancy. J Intractable Dis. 2021;20:5.

[8] Perovic M Gojnic M Arsic B. Relationship between mid-trimester ultrasound fetal liver length measurements and gestational diabetes mellitus. J Diabetes. 2015;7:497–505. 25124095 10.1111/1753-0407.12207

[9] Xie C Man Q Wan X. The clinical value of combining shear wave elastography, VOCAL technique, and T2* MRI of early gestation placenta to predict pre-eclampsia. J Clin Ultrasound. 2024;52:13–9. 37883126 10.1002/jcu.23575

[10] Ridder A O’Driscoll J Khalil A. Routine first-trimester pre-eclampsia screening and maternal left ventricular geometry. Ultrasound Obstet Gynecol. 2024;63:75–80. 37448160 10.1002/uog.26306

[11] Fujita Y Nakanishi TO Sugitani M. Placental elasticity as a new non-invasive predictive marker of pre-eclampsia. Ultrasound Med Biol. 2019;45:93–7. 30342781 10.1016/j.ultrasmedbio.2018.09.007

[12] Sugitani M Fujita Y Yumoto Y. A new method for measurement of placental elasticity: acoustic radiation force impulse imaging. Placenta. 2013;34:1009–13. 24075540 10.1016/j.placenta.2013.08.014

[13] Yang Y Zeng M Niu JM. Evaluation of placental function in patients with gestational hypertension syndrome with spectral Doppler ultrasound combined with three-dimensional energy Doppler ultrasound. Oncol Imaging. 2019;28:5.

[14] Han L Gao YH Wang ZF. Diagnostic value of three-dimensional energy Doppler for placental implantation in late pregnancy. J Clin Pathol. 2016;36:1616–9.

[15] Neto RM Ramos JG. 3D power Doppler ultrasound in early diagnosis of preeclampsia. Pregnancy Hypertens. 2016;6:10–6. 26955765 10.1016/j.preghy.2015.11.003

[16] Ge CX Guo JF. Quantitative analysis of placental elasticity in patients with pre-eclampsia by shear wave elastography. J Clin Ultrasound Med. 2019;21:855–7.

[17] Kiliç F Kayadibi Y Yüksel MA. Shear wave elastography of placenta: in vivo quantitation of placental elasticity in preeclampsia. Diagn Interv Radiol. 2015;21:202–7. 25858523 10.5152/dir.2014.14338 PMC4463268

[18] Anuk AT Tanacan A Erol SA. Value of shear-wave elastography and cerebral-placental-uterine ratio in women diagnosed with preeclampsia and fetal growth restriction in prediction of adverse perinatal outcomes. J Matern Fetal Neonatal Med. 2022;35:10001–9. 35647897 10.1080/14767058.2022.2081804

[19] Djokic V Jankovic S Labudovic-Borovic M. Pregnancy-induced hypertension decreases K(v)1.3 potassium channel expression and function in human umbilical vein smooth muscle. Eur J Pharmacol. 2020;882:173281. 32562800 10.1016/j.ejphar.2020.173281

[20] Yang Z Zhang WY. Interpretation of guidelines for the diagnosis and treatment of hypertension during pregnancy (2015). Chin J Pract Gynecol Obstet. 2015;31:886–93.

[21] McCarthy R Orsi NM Treanor D. Three-dimensional digital reconstruction of human placental villus architecture in normal and complicated pregnancies. Eur J Obstet Gynecol Reprod Biol. 2016;197:130–5. 26745392 10.1016/j.ejogrb.2015.12.015

[22] Roland CS Hu J Ren CE. Morphological changes of placental syncytium and their implications for the pathogenesis of preeclampsia. Cell Mol Life Sci. 2016;73:365–76. 26496726 10.1007/s00018-015-2069-x PMC4846582

[23] Chen XQ Liang BR. Predictive value of placental volume and vascular index in the second trimester of pregnancy by three-dimensional ultrasound in the prediction of recurrent preeclampsia. Chin J Fam Plan. 2020;28:5.

[24] Cui JL Wang YH. Research progress on the role of microparticles in regulating the expression of neutrophil extracellular trapping nets in the pathogenesis of preeclampsia. Int J Obstet Gynecol. 2020;47:373–7.

